# A Short Synthesis of (±)‐3‐Demethoxyerythratidinone by Ligand‐Controlled Selective Heck Cyclization of Equilibrating Enamines

**DOI:** 10.1002/anie.201701775

**Published:** 2017-05-04

**Authors:** Emma E. Blackham, Kevin I. Booker‐Milburn

**Affiliations:** ^1^ School of ChemistryUniversity of BristolCantock's CloseBristolBS8 1TSUK

**Keywords:** cycloaddition, Heck reaction, ligands, photochemistry, total synthesis

## Abstract

A short, 5‐step total synthesis of (±)‐3‐demethoxyerythratidinone from a simple pyrrole derivative is described. Features include the formation of gram quantities of a key tricylic aziridine from a challenging photochemical cascade reaction through the use of flow photochemistry. The final step involved a highly unusual Heck cyclization whereby ligand control enabled efficient formation of the natural product in 69 % yield from the minor isomer present in an equilibrating mixture of labile enamines.

The alkaloid (+)‐3‐demethoxyerythratidinone **1** is one of over 100 natural products produced by the *Erythrina* genus of flowering plants.^1^ The *Erythrina* alkaloids display a broad range of pharmacological activities including hypotensive, sedative, neuromuscular blocking, CNS depressing and curare‐like activities. The key structural feature of this family is the tetracyclic tetrahydroisoquinoline core. Since the first total synthesis of **1** by Tsuda in 1984,^2^ this tetracyclic alkaloid has been used by others in order to demonstrate the utility of various synthetic methodologies.^3^ A very elegant synthesis was recently reported by Reisman, where chiral sulfinyl imine chemistry was used to control the stereochemistry of the key quaternary center,3d giving (−)‐3‐demethoxyerythratidinone in just six steps overall (Figure 1).

**Figure 1:**
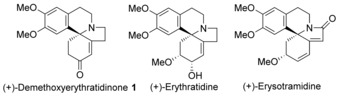
Members of the *Erythrina* family of alkaloids.

Individually both photochemical and Pd‐catalyzed cross‐coupling methodologies have been shown to be powerful techniques in organic synthesis as well as valuable tools in the synthesis of complex molecular architectures.^4^ We have previously reported the photochemical transformation of simple *N*‐butenyl‐substituted pyrroles into complex tricyclic aziridines **2**.^5^ We recently demonstrated that these strained photochemical products undergo a range of thermal and Pd‐catalyzed ring‐opening/annulation reactions to produce a broad range of fused polyheterocycles, in just two steps from simple pyrroles (Scheme 1).^6^ We were therefore keen to exploit the functionality and inherent strain in these aziridines as part of an alkaloid synthesis, in particular the aziridine carboxylates **3**. Herein we report a short total synthesis of (±)‐**1** utilizing a highly unusual and selective, ligand controlled intramolecular Heck‐reaction onto one of a pair of equilibrating enamine intermediates.

**Scheme 1:**
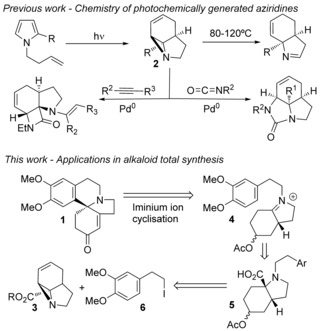
Synthesis and reactivity of tricyclic aziridines and initial retrosynthetic analysis of 3‐demethoxyerythratidinone (**1**).

Our initial strategy to **1** (Scheme 1) involved aryl cyclization onto the iminium ion **4** which itself would be generated by in situ decarboxylation of the amino acid **5**.^7^ Although iminium ion cyclizations are one of the most frequently used approaches to such alkaloids,^8^ these have usually involved the intermediary of *N*‐acyliminium ions.^9^ The requisite amino‐acid **5** should be obtained by Pd^0^‐catalyzed acetate ring‐opening of the aziridine **3** followed by *N*‐alkylation with the iodide **6**.

Irradiation of pyrrole **7** (254 nm) gave the aziridine (±)‐**8** in a 39 % yield (Scheme 2). As before, we found that this two‐photon process suffers from low productivity due to a likely low overall quantum yield and as such was limited to ca. 60 mg quantities of (±)‐**8** at a time using a 6 W batch reactor.^5^ Fortunately, we found that using our three‐lamp FEP‐flow reactor gave routine access to 1.91 g of aziridine (±)‐**8** in a single 373 min run (productivity of 7.37 g/24 h). This highlights once again the value of flow photochemistry for scaling‐up high dilution/low quantum yield reactions—this would simply not be possible on laboratory batch scale.^10^ We then investigated the Pd‐catalyzed Tsuji–Trost type reaction of (±)‐**8** with acetate as nucleophile, this proceeded in excellent yield (81 %) to give a single diastereomer of the (S_N_2′) ring‐opened amine (±)‐**9**. It was interesting to observe that after 3 h the regioisomeric ratio (NMR) of S_N_2′ to S_N_2 ring‐opened product was 1:15. However, after 16 h none of the S_N_2 ring opened product remained, indicating that this reaction is under thermodynamic control via the initially formed S_N_2 product. The diastereomer obtained is in agreement with the classic “double‐inversion” Tsuji–Trost mechanism for soft nucleophiles, where a net retention of stereochemistry is observed.^11^

**Scheme 2:**
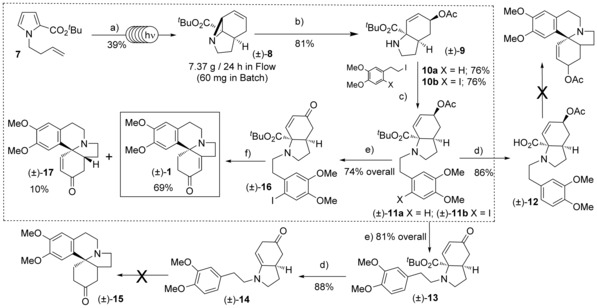
Total synthesis of (±)‐3‐demethoxyerythratidinone. Reagents and conditions: a) *hν*, 254 nm, 3×36 W, FEP flow reactor, cyclohexane; b) Pd_2_(dba)_3_, (3 mol %) P(OPh)_3_ (25 mol %), AcOH (5 equiv), CH_2_Cl_2_, RT, 16 h; c) K_2_CO_3_, TBAI, MeCN, 90 °C, 1 h; d) TFA, CH_2_Cl_2_, RT, 16 h; e) K_2_CO_3_, MeOH/H_2_O, 60 °C, 2–4 h, then TPAP, NMO, CH_2_Cl_2_, 4 Å sieves, RT, 16 h; f) TFA, CH_2_Cl_2_, RT, 16 h then Pd(OAc)_2_ (20 mol %), PCy_3_ (40 mol %), K_2_CO_3_ (1.5 equiv), toluene, 110 °C, 16 h.

Amine (±)‐**9** was then alkylated with the iodide **10 a** (X=H) in good yield using phase transfer conditions. The *tert*‐butyl group in (±)‐**11 a** was cleaved in 86 % yield by treatment with TFA to give the amino‐acid (±)‐**12**. In an attempt to promote the key iminium ion cyclization (cf. **4** to **5**) the acid (±)‐**12** was subjected to a number of decarboxylation conditions,^7^, ^12^ including both POCl_3_ and oxalyl chloride with catalytic DMF in CH_2_Cl_2_. In all instances the reaction was unsuccessful and no product could be detected amongst the complex reaction mixtures obtained. As it was suspected that this may have been related to elimination of the acetate group, a telescoped sequence was investigated, where (±)‐**11 a** was hydrolyzed and the resultant alcohol oxidized to the enone (±)‐**13** with TPAP (74 % over two steps). In an attempt to access the desired amino acid, *tert*‐butyl ester (±)‐**13** was treated with TFA. However, upon cleavage of the *tert*‐butyl ester the resultant acid underwent spontaneous decarboxylation to generate the isolable, but labile enamine (±)‐**14** in excellent yield. This is clearly a result of the conjugated enone system enabling (±)‐**13** to behave as a vinylogous β‐ketoester, sensitive to decarboxylation under acid conditions. Under acidic conditions it was postulated that (±)‐**14** should be in equilibrium with the desired iminium species (cf. **4**). However, despite further investigation no cyclization to (±)‐**15** was observed when (±)‐**14** was treated with a range of acid catalysts including PPA, *p*‐toluene sulfonic acid, TFA and camphor sulfonic acid.^13^

Intrigued by the ease of the decarboxylation of (±)‐**13** to (±)‐**14**, we elected to change our strategy and focus on an approach involving a Heck cyclization onto an enamine to generate the quaternary spirocyclic centre of (±)‐**1**.^14^, ^15^ Such a strategy should generate a high enough quantity of the isomeric natural product (±)‐**17** to convert it to (±)‐**1** by a reduction/ dehydrogenation sequence.

Treatment of (±)‐**9** with **10 b** gave the aryl iodide (±)‐**11 b**. Hydrolysis of the acetate and oxidation with TPAP gave the enone (±)‐**16** in 74 % overall yield. Cleavage/decarboxylation of (±)‐**16** gave the enamines (±)‐**18** and **19,** which although labile to isolation and separation, were formed in essentially quantitative yield as a 4:1 mixture, respectively. Initial attempts at the Heck cyclization were carried out on this mixture using Pd^0^/DMF conditions described by Waldmann^16^ and led to a 65 % yield of (±)‐**17** as a single diastereomer (Table 1, entry 1). However, during reaction optimization a peculiar observation was noted when conditions described by Orito^17^ were employed (Table 1, entry 2). This gave not only (±)‐**17** (56 %) but also the natural product (±)‐**1** in 28 % isolated yield. Under these conditions it was likely that Heck cyclization of **19** to (±)‐**1** was occurring to a significant degree.

**Table 1:** Optimization of an intramolecular Heck reaction via equilibrating enamine isomers and the effect of phosphorous ligands. 
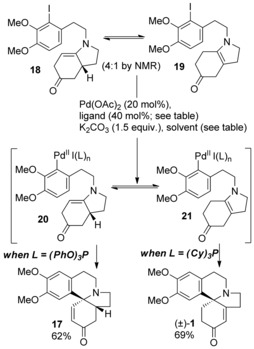

Entry	Catalyst	Ligand	Solvent	(±)‐**17** [%]^[b]^	(±)‐**1** [%]^[b]^
1^[a]^	Pd_2_(dba)_3_	PPh_3_	DMF	65	0
2	Pd(OAc)_2_	PPh_3_	PhMe	56	28
3	Pd(OAc)_2_	P(OPh)_3_	PhMe	62	4
4	Pd(OAc)_2_	P(4‐MeOC_6_H_4_)_3_	PhMe	13	53
**5**	**Pd(OAc)_2_**	**P(Cy)_3_**	**PhMe**	**<10**	**69**
6	Pd(OAc)_2_	P(*t*Bu)_3_	PhMe	8	3
7	Pd(OAc)_2_	(−)‐DIOP	PhMe	55	40^[c]^
8	Pd(OAc)_2_	(*R*)‐^*i*^Pr‐PHOX	PhMe	30	32^[d]^

[a] 1 equiv of TBAI. [b] Isolated yields. [c] 25 % *ee* in favor of (−)‐**1**. [d] 38 % *ee* in favor of (+)‐**1**.

This raised the attractive prospect of modifying the reaction conditions such that (±)‐**1** could be produced as the sole product from the minor component (**19**) by in situ equilibration of this enamine mixture, thus considerably shortening the overall synthesis of this natural product. We postulated that varying the Pd ligand might affect reaction selectivity and so a brief survey was undertaken (Table 1).

Use of triphenylphosphite had the opposite effect giving 62 % (±)‐**17** with only 4 % (±)‐**1** (entry 3). Employing the more electron‐rich tris(*p*‐methoxyphenyl) phosphine as a ligand, however, favored formation of (±)‐**1** (53 %) over (±)‐**17** (13 %) (entry 4). The most consistent results were obtained with the alkyl phosphine ligand (Cy)_3_P which gave 69 % isolated yield of **1** and just 10 % of the isomer (±)‐**17** (entry 5) in a single telescoped sequence from the ester (±)‐**16**. This optimized result concluded a 5‐step synthesis of (±)‐**1** in 15 % overall yield from pyrrole **7** (Scheme 2).

It is clear that electron‐rich phosphines (entries 4 and 5) likely favor the formation of (±)‐**1** by cyclization of the organopalladium‐enamine isomer **21**. Conversely, comparatively electron poor ligands (entries 2 and 3) likely favor cyclization to (±)‐**17** via the isomer (±)‐**20**. It is possible that L_*n*_HPdI from β‐hydride elimination may serve as a convenient catalyst for the isomerization of (±)‐**18** to **19** and different ligands will affect the reactivity^18^, ^19^ of such a catalyst e.g. reductive elimination vs. enamine isomerization. Waldmann^16^ previously observed isomerized products from Heck cyclization onto dihydro‐4‐pyridones (enaminones) and attributed these to isomerization of initially formed α‐palladio‐ketones via σ‐π‐σ‐allyl rearrangement. In our case the same reaction conditions (Table 1, entry 1) lead only to (±)‐**17** and so it is likely that a pathway involving isomerization of (±)‐**18** and **19** is plausible.

As **19** is achiral then this opened up the possibility of effecting an asymmetric synthesis of (+)‐**1** directly from the mixture of enamines. After screening a range of chiral ligands it became clear that most resulted in a mixture of (±)‐**17** and **1** with little *ee* observed for the latter (see the Supporting Information). Use of (−)‐DIOP gave 55 % yield of (±)‐**17** and 40 % of **1** with an *ee* of 25 % in favor of (−)‐**1** (Table 1, entry 7). (*R*)‐^*i*^Pr‐PHOX (Table 1, entry 8) gave a 32 % isolated yield of **1** with an *ee* of 38 % in favor of the natural enantiomer (+)‐**1**. Attempts to increase this by use of additives (e.g. Ag salts) resulted in inferior results or inhibition of reaction.

In conclusion, we have developed a short 5‐step sequence to (±)‐3‐demethoxyerythratidinone in 15 % overall yield from the simple pyrrole carboxylate **7**. Notable features include the use of a powerful two‐photon cycloaddition–rearrangement reaction to provide a reactive aziridine **8**, the key intermediate of the synthesis. This was produced in gram quantities using flow‐photochemistry, which would have been very difficult to achieve in batch due to the high dilution and irradiation times required. This study also uncovered a highly unusual and selective, ligand‐controlled intramolecular Heck reaction. By use of electron rich phosphines (±)‐3‐demethoxyerythratidinone (**1**) was formed as the *major* product from Heck cyclization onto the *minor* component of a pair of enamine isomers. Use of electron poor phosphine ligands gave an isomer of the natural product by cyclization onto the major enamine isomer in the mixture. The generality of such a switching process and the mechanistic understanding merits further investigation. Use of chiral ligands for the asymmetric synthesis of (+)‐**1** yielded mixed results; up to 38 % *ee* was observed in favor of (+)‐**1** but at the expense of product selectivity.

## Conflict of interest

The authors declare no conflict of interest.
